# Novel inhibitory brainstem neurons with selective projections to spinal lamina I reduce both pain and itch

**DOI:** 10.1002/cne.25076

**Published:** 2020-12-04

**Authors:** Lindsay J. Agostinelli, Alexander G. Bassuk

**Affiliations:** ^1^ Department of Neurology Roy J. and Lucille A. Carver College of Medicine, The University of Iowa Iowa City Iowa USA; ^2^ Department of Pediatrics University of Iowa Iowa City Iowa USA

**Keywords:** activation, anterograde tracing, capsaicin, chemogenetic, dynorphin, histamine, lamina I, LJA5, prodynorphin, RRID AB_11180610, RRID: AB_11213126, RRID: AB_262156, RRID:AB_2631318, stimulation

## Abstract

Sensory information is transmitted from peripheral nerves, through the spinal cord, and up to the brain (“bottom up” pathway). Some of this information may be modulated by “top‐down” projections from the brain to the spinal cord. Discovering endogenous mechanisms for reducing pain and itch holds enormous potential for developing new treatments. However, neurons mediating the top‐down inhibition of pain are not well understood, nor has any such pathway been identified for itch sensation. Here, we identify a novel population of GABAergic neurons in the ventral brainstem, distinguished by prodynorphin expression, which we named LJA5. LJA5 neurons provide the only known inhibitory projection specifically to lamina I of the spinal cord, which contains sensory neurons that transmit pain and itch information up to the brain. Chemogenetically activating LJA5 neurons in male mice reduces capsaicin‐induced pain and histamine‐induced itch. Identifying this new pathway opens new treatment opportunities for chronic, refractory pain, and pruritis.

## Graphical abstract

We identified a novel population of inhibitory prodynorphin neurons in the brainstem which we named LJA5. LJA5 neurons selectively target lamina I of the spinal cord, and other sensory nuclei including the lateral parabrachial and periaqueductal gray. Activation of LJA5 neurons reduces pain and itch.

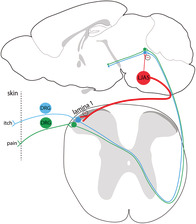

## 1 INTRODUCTION

Chronic pain and itch afflict millions of patients, and current treatments are largely ineffective (Mansfield et al., 2016; Yosipovitch & Bernhard, 2013). We do not fully understand the neural control of pain and itch, and are thus limited in developing therapies targeting the underlying circuitry in the brain or spinal cord.

Current models show that lamina I of the spinal cord contains the neurons that transmit pain and itch information from the skin up to the brain (Andrew & Craig, 2001; Craig, 2002; Dong & Dong, 2018). Most of what we know about the neural control of pain and itch involves this bottom‐up pathway. Many studies have elucidated mechanisms by which the peripheral nervous system first detects pain and itch, then relays this information from skin to spinal cord (Caterina et al., 2000; Duan et al., 2014; LaMotte et al., 2014; Liu et al., 2009; Sun et al., 2009). However, it is not well understood how the brain modulates pain and itch sensations in a top‐down fashion.

The simplest and most direct way for the brain to suppress pain and itch would be a direct, inhibitory projection to lamina I sensory neurons, but previously identified projections do not specifically target lamina I. The idea that our brains can suppress unpleasant sensations began with a report in 1969 that electrically stimulating the brainstem in rats could produce a surgical level of anesthesia, without anesthetic drugs (Reynolds, 1969). A roadblock to developing this observation is that the stimulated region of the brainstem does not send any inhibitory projections to the spinal cord. Prior investigators have focused on brainstem regions that project axons nonspecifically targeting many laminae of the spinal cord (Basbaum et al., 1978; Fields et al., 1991; Millan, 2002). In addition to top‐down inhibition of pain, pharmacological studies suggest the possibility of top‐down itch control, but this has no known neural substrate (Gotoh et al., 2011; Zhao et al., 2014). Therefore, the specific neural pathway the brain uses to modulate pain remains incompletely understood, and whether the brain can inhibit itch in a top‐down fashion is unknown.

We discovered a novel group of inhibitory neurons in the ventrolateral brainstem that sends dense axonal projections specifically to lamina I of the spinal cord (Agostinelli et al., 2020). Given that lamina I neurons transmit pain, temperature, and itch (Craig, 2002; Dong & Dong, 2018), we hypothesized that these neurons inhibit those sensory modalities. To test this hypothesis, we used a genetically targeted, excitatory designer receptor exclusively activated by designer drug (DREADD) to test the ability of these neurons to reduce pain, itch, and temperature sensation.

## 2 MATERIALS AND METHODS

Note: All experiments and analysis were completed before March 2020.

### 2.1 Animals

To generate prodynorphin reporter mice, we crossed *Pdyn‐IRES‐Cre* mice with R26‐lsl‐L10‐green fluorescent protein (GFP) mice, two lines of mice that were produced and characterized in the Lowell lab and were published previously (Geerling et al., 2016; Krashes et al., 2014). *Pdyn*‐*IRES‐*Cre mice are available from The Jackson Laboratory (stock No. 027958). *Pdyn*‐*IRES‐*Cre mice selectively express Cre recombinase in cells that have expressed prodynorphin, and L10‐GFP mice have a loxP‐flanked STOP cassette which prevents the transcription of downstream GFP. We crossed these mice with *Pdyn‐IRES‐Cre* mice because in the presence of Cre recombinase, the STOP cassette is deleted, resulting in offspring that express GFP selectively in Cre‐expressing dynorphin cells, which we abbreviate as *Pdyn*‐GFP.

We housed all mice on a 12:12 light:dark cycle with lights on at 6:00 a.m. at 22°C ambient temperature with ad libitum access to food and water. All protocols and care of the mice followed National Institute of Health guidelines and were approved by the University of Iowa Institutional Animal Care and Use Committee.

### 2.2 Surgery/microinjections

We used Cre‐conditional anterograde tracing to map projections of LJA5 neurons and Cre‐conditional DREADDs to activate LJA5 neurons. We injected the LJA5 region of male Cre‐expressing mice (10–11 weeks old) with an adeno‐associated viral vector (AAV8‐hEF1α‐DIO‐synaptophysin‐mCherry, 2.5 × 10^13^ vg/ml, developed by Dr Rachael Neve at the Massachusetts Institute of Technology McGovern Institute for Brain Research Viral Vector Core) coding for Cre‐dependent synaptophysin tagged with the red fluorescent protein mCherry (AAV‐DIO‐synaptophysin‐mCherry) (*n* = 4 mice) or an adeno‐associated viral vector (AAV8‐hSyn‐DIO‐hM3D(Gq)‐mCherry, 4.6 × 10^12^ vg/ml, UNC Vector Core) coding for Cre‐dependent hM3Dq excitatory DREADD (hM3Dq) tagged with the red fluorescent protein mCherry (AAV‐DIO‐hM3Dq‐mCherry) (*n* = 26, male mice). In these AAVs, the synaptophysin‐mCherry or hM3Dq‐mCherry sequence is inverted and surrounded by pairs of loxP and lox2722 sites, thus limiting synaptophysin‐mCherry or hM3Dq‐mCherry expression to cells that contain Cre recombinase. By injecting this AAV into mice that express Cre recombinase selectively in prodynorphin‐expressing neurons, we restricted synaptophysin‐mCherry or hM3Dq‐mCherry expression to LJA5 dynorphinergic neurons.

For stereotaxic delivery of these AAVs, we first anesthetized mice with ketamine/xylazine (100/10 mg/kg. i.p.) and unilaterally microinjected 90 nl of AAV‐DIO‐synaptophysin‐mCherry into the right side of the hindbrain in the LJA5 region (coordinates from bregma: AP ‐4.83 mm, RL 1.35 mm, DV ‐5.50 mm from the top of the skull). We injected AAV‐DIO‐hM3Dq‐mCherry bilaterally, using the same coordinates.

Four weeks after stereotaxic injection, we perfused the mice with AAV‐DIO‐synaptophysin‐mCherry, and performed behavioral experiments on the mice with AAV‐DIO‐hM3Dq‐mCherry before perfusion. For perfusions, we deeply anesthetized mice with ketamine/xylazine (150/15 mg/kg i.p.) and transcardially perfused them with 50 ml phosphate‐buffered saline (PBS; pH 7.4) followed by 50 ml of buffered 10% formalin (pH 7.0; Fisher Scientific, Fair Lawn, NJ). We removed and postfixed the brains and spinal cords for 12 h in 10% formalin and then cryoprotected them in PBS containing 20% sucrose. We later sectioned the brains and spinal cords at 30 μm into a 1:4 series on a freezing microtome.

### 2.3 Histology

All tracing and DREADD experimental brains were processed as described above. We perfused *n* = 4 additional *Pdyn‐*GFP mice without stereotaxic microinjections for immunohistology and RNAscope in situ hybridization. We cut one brain into sagittal sections to study the relationship of LJA5 to other neuronal populations. After overnight incubation in rabbit anti‐tyrosine hydroxylase (TH) IgG (1:2000; Millipore; AB152; RRID: AB_11213126), goat anti‐ChAT IgG (1:500; Millipore AB144P; RRID: AB_262156, goat, polyclonal), and chicken anti‐GFP IgG (1:5000; Invitrogen; A10262; RRID AB_11180610), we placed sagittal sections in donkey anti‐rabbit IgG conjugated to Alexa Fluor 555 (1:500; Invitrogen; A31572), donkey anti‐chicken IgG conjugated to Alexa 488 (1:500; Jackson ImmunoResearch; 703‐065‐155), and biotinylated donkey anti‐goat IgG (1:500, Jackson ImmunoResearch, 705‐065‐147) for 1 h, followed by PBS washes and then Cy5‐conjugated streptavidin (1:1000; Invitrogen; SA1001) for 1 h.

We labeled the injection sites of *Pdyn‐*GFP mouse brains using double immunofluorescence. After overnight incubation in rat anti‐mCherry IgG (1:2000; Life Technologies; M112171, AB_2536611) and chicken anti‐GFP IgG, we placed sections in donkey anti‐rat IgG conjugated to Cy3 (1:500; Jackson ImmunoResearch 712‐165‐153) and donkey anti‐chicken IgG. Brains injected with AAV‐DIO‐hM3Dq‐mCherry were also stained overnight with rabbit anti‐Fos IgG (1:10,000; Millipore; ABE457; RRID:AB_2631318) followed by 1 h in biotinylated donkey anti‐rabbit IgG secondary antiserum (1:500; Jackson ImmunoResearch, 711‐065‐152) and 1 h of Cy5‐conjugated streptavidin.

We then stained a series of AAV‐DIO‐synaptophysin‐mCherry injected brains with DAB. We placed sections into 0.3% hydrogen peroxide for 30 min and then incubated them overnight in rat anti‐mCherry IgG (1:5000). We then placed sections in biotinylated donkey anti‐rat IgG secondary antisera (1:500; Jackson ImmunoResearch; 712‐066‐153) followed by 1 h in avidin–biotin complex (Vectastain ABC Elite Kit; Vector Laboratories, Burlingame, CA). We labeled mCherry black with DAB in tris‐buffered saline containing 0.024% hydrogen peroxide and 0.2% ammonium nickel (II) sulfate (Sigma).

We stained a series of spinal cord sections from AAV‐DIO‐synaptophysin‐mCherry injected brains with dorsal root ganglia markers. We incubated sections overnight with rat anti‐mCherry, rabbit anti‐CGRP IgG (1:10,000; Penninsula; T‐4032), and isolectin‐B4 (IB4) conjugated to Alexa 647 (1:100; Life Technologies; 132450). The next day we incubated sections for 1 h in donkey anti‐rabbit IgG secondary antisera (1:500; Jackson ImmunoResearch; 703‐065‐155) followed by 1 h of Cy5‐conjugated streptavidin (Table 1).

**TABLE 1:** Details on antisera used in these experiments

Antigen	Immunogen	Manufacturer, catalog or lot number, the RRID species, mono or poly	Concentration
ChAT	Human placental enzyme	Millipore, Cat# AB144P, RRID: AB_262156, goat, polyclonal	1:2000 for DAB, 1:1000 for fluorescence
fos	KLH‐conjugated linear peptide corresponding to the N‐terminus of human c‐Fos	1:10,000; Millipore; ABE457; RRID:AB_2631318; rabbit, polyclonal	1:10,000
GFP	GFP from *Aequorea victoria*	Life Technologies, A10262, RRID AB_11180610, chicken, polyclonal	1:5000
mCherry	Full length protein m‐Cherry	Life Technologies/Invitrogen, Cat# M11217, RRID AB_2536611, rat, monoclonal	1:5000 for DAB, 1:2000 for fluorescence
TH	Native TH from rat pheochromocytoma	Millipore, sheep polyclonal, Cat# AB1542, RRID: AB_11213126	1:2000

Abbreviations: GFP, green fluorescent protein; TH, tyrosine hydroxylase.

After immunolabeling, we mounted and dried sections on Superfrost Plus slides. For sections with fluorescence immunolabeling, we coverslipped with fade‐retardant mounting medium containing DAPI (Vectashield; Vector Labs). For DAB‐labeled tissue, we dehydrated sections in graded ethanols and cleared in xylenes before coverslipping with a toluene‐based mounting media (Cytoseal; Thermo Scientific). We acquired whole‐slide images using a slide‐scanning microscope (Olympus VS120) and reviewed slides in OlyVIA software (Olympus).

### 2.4 Validation of neurochemical specificity

*Pdyn‐*GFP mice were described previously (Geerling et al., 2016; Krashes et al., 2014). To confirm that in the LJA5 region specifically, GFP expression was limited to prodynorphin‐expressing neurons, we performed in situ hybridization with RNAscope using a probe directed against mouse *Pdyn* (318771) and the Advanced Cell Diagnostics RNAscope multiplex V1 fluorescent assay.

We additionally labeled *Pdyn‐*GFP tissue for GABAergic and glutamatergic genetic markers. We used probes directed against mouse Vgat/*Slc32a (*319191), *Gad1* (400951), Vglut2/*Slc17a6* (319171) (Advanced Cell Diagnostics).

### 2.5 Analysis of injection sites

To measure the specificity of neuronal transduction, we immunostained sections for GFP (to enhance the native GFP of reporter neurons) and for mCherry, then counted the number of GFP+ cells that expressed mCherry across all sections containing LJA5 neurons (spanning typically six axial sections from a 1:4 series through the rostral hindbrain). Three mice injected with AAV‐DIO‐hM3Dq‐mCherry had missed injection sites (only 10 or fewer mCherry+ cells) and were thus excluded from the study.

Analysis of AAV‐DIO‐synaptophysin‐mCherry injection sites revealed that 97.9% of LJA5 GFP+ cells expressed mCherry. All mCherry cells were colocalized with GFP.

For AAV‐DIO‐hM3Dq‐mCherry, mCherry was expressed in a mean of 49.3% ± 4.6 of LJA5 GFP+ neurons (range 30.2–75.6%).

### 2.6 Bouton counting of anterograde tracing

To estimate the relative density of LJA5 projections to efferent targets, we counted LJA5 boutons in each region. We used AAV‐DIO‐synaptophysin‐mCherry cases because the mCherry (fused to a synaptically localized protein) robustly labels axon terminals and *en passant* synaptic boutons. We imaged whole brain and spinal cord sections with a slide‐scanning microscope (Olympus VS120), using a ×40 objective and extended focal imaging (EFI). EFI takes a z‐series of images through the tissue and combines them into one in‐focus image. We loaded images of selected brain regions into Adobe Illustrator, and using the symbols tool, quantified the number of boutons in a given brain region. We quantified boutons in two sections containing the PAG terminal field, three sections through the PB, and five spinal cord sections (cervical, upper thoracic, lower thoracic, lumbar, and sacral levels). We added the bouton counts from sample sections through each region and multiplied by the total number of sections that contain the axon‐terminal field in that region. Then, to estimate the relative proportion of boutons, we divided this number by the estimated total number of boutons across all three sample regions (PAG, PB, and spinal cord).

### 2.7 DREADD behavior experiments

Behavior experiments were performed in male mice. Three weeks after stereotaxic microinjection of AAV‐DIO‐hM3Dq‐mCherry, mice (14 weeks of age) were habituated to testing chambers, handling, and i.p. injections. One week later, we began behavior testing. Testing consisted of an i.p. injection of saline or clozapine‐N‐oxide (CNO) (1.0 mg/kg, 0.1 ml/10 g diluted in 0.9% saline) 30 min prior to testing. Tests were conducted in the light phase at approximately the same circadian time each day (ZT4 ~ 10:00 am). Each mouse was tested in a given experiment twice, and received both saline and CNO in a randomized order. All behavior scoring was completed by research assistants blinded to the experimental conditions.

### 2.8 Pain

Our testing chamber consisted of a clear glass floor with a camera below, and plexiglass dividers to separate the mice. Mice were habituated to the chamber for 30 min on three separate days. On test days, mice received either saline or CNO and were placed in the chamber for 30 min of habituation. We then injected 1 μg capsaicin in a volume of 10 μl into the plantar surface mice of the hindpaw (0.1% capsaicin dissolved in saline with 5% ethanol and 5% tween 80, randomized injections into left vs right hindpaw). The mice were returned to the Hargreaves apparatus and recorded from below for 15 min. The amount of time spent licking and flinching the paw was scored. After 8 days, mice were given saline or CNO and then tested again with a capsaicin injection in the opposite hindpaw.

### 2.9 Mechanical hypersensitivity testing

One hour after the capsaicin injection (90 min after the i.p. injection of saline or CNO), we placed mice on a wire mesh floor for 15 min of habituation. Then we performed a common test for mechanical hypersensitivity, the von Frey test (Chaplan et al., 1994). We applied graded mechanical pressure using calibrated nylon monofilaments (von Frey filaments) from below the wire floor to the hindpaws of the mice. This application of low‐pressure von Frey filaments is innocuous, but if the animal experiences discomfort it will withdraw its paw (with a brisk flexion movement). We applied a series of increasingly stiff von Frey filaments (each filament applied eight times) to the plantar surface of the hindpaw, from below, until reaching a threshold pressure, defined as greater than or equal to 50% hindpaw withdraws for a given filament.

### 2.10 Itch

At least 2 days before testing, we shaved a patch of hair off the nape of each mouse. On testing days, mice received either saline or CNO 30 min before testing, and were placed in the recording chamber for these 30 min for habituation. Intradermal (i.d.) injections of histamine (500 μg in 50 μl saline) were made into the nape of the neck, producing a 3 mm intradermal bleb. One trial each was excluded from two mice, then repeated, due to a subcutaneous injection (no intradermal bleb). Mice were video recorded for 40 min. Scratching bouts were scored in 5‐min bins for 40 min. A scratching bout was defined as the animal lifting its hindpaw to the neck for any number of scratches/swipes before returning the hindpaw to the floor.

### 2.11 Hargreaves/radiant heat

Animals were first placed on the Hargraves apparatus (IITC Life Science, Model 400) for three, 30‐min sessions on separate days for habituation. Then, on test days, mice received an injection of saline or CNO and were placed on the Hargraves apparatus for 30 min. Using a beam intensity of 30%, we quantified the latency to hindpaw withdrawal.

### 2.12 Temperature preference

Our test chamber consisted of two adjacent plates (Columbus Instruments) connected by an acrylic bridge between plates. For habituation, both plates were kept at 30°C, and mice were habituated to this setup for 15 min on three separate days. For testing, one plate was always kept at 30°C (preferred temperature for mice when presented with a gradient of choices (Vriens et al., 2011)) referred to as “Plate A.” The other plate (“Plate B") was either 15, 30, or 45°C, presented in a randomized order on different days. The orientation of Plates A and B was also randomized on the left or the right so that mice did not develop a plate preference. On testing days, mice received an i.p. injection of either saline or CNO 30 min before testing. Then mice were placed on the acrylic bridge and recorded for 10 min. Videos were scored for the amount of time that the animal spent on Plate B.

### 2.13 Ambulation

To measure mouse activity after CNO and saline i.p. injections, we used computer analysis of 10‐min temperature preference video recordings when both Plates A and B set to the same preferred temperature (30°C). We used Ethovision software (Noldus) to calculate the total distance traveled and time spent moving.

### 2.14 Statistical analysis

Data are presented as mean ± *SEM*. *n* represents the number of mice analyzed. Most statistical comparisons were conducted by two‐tailed, paired Student's *t* test. We used two‐way analysis of variance with Holm–Sidak's multiple comparisons test to compare histamine‐induced responses. Power analysis was used to determine sample size using pilot histamine‐induced scratching data and capsaicin‐induced pain behavior; with an alpha of .05 and power of 0.8, the projected sample sizes were *n* = 8 and *n* = 9, respectively.

## 3 RESULTS

### 3.1 LJA5 inhibitory brainstem neurons selectively project to lamina I of the entire spinal cord

**FIGURE 1:**
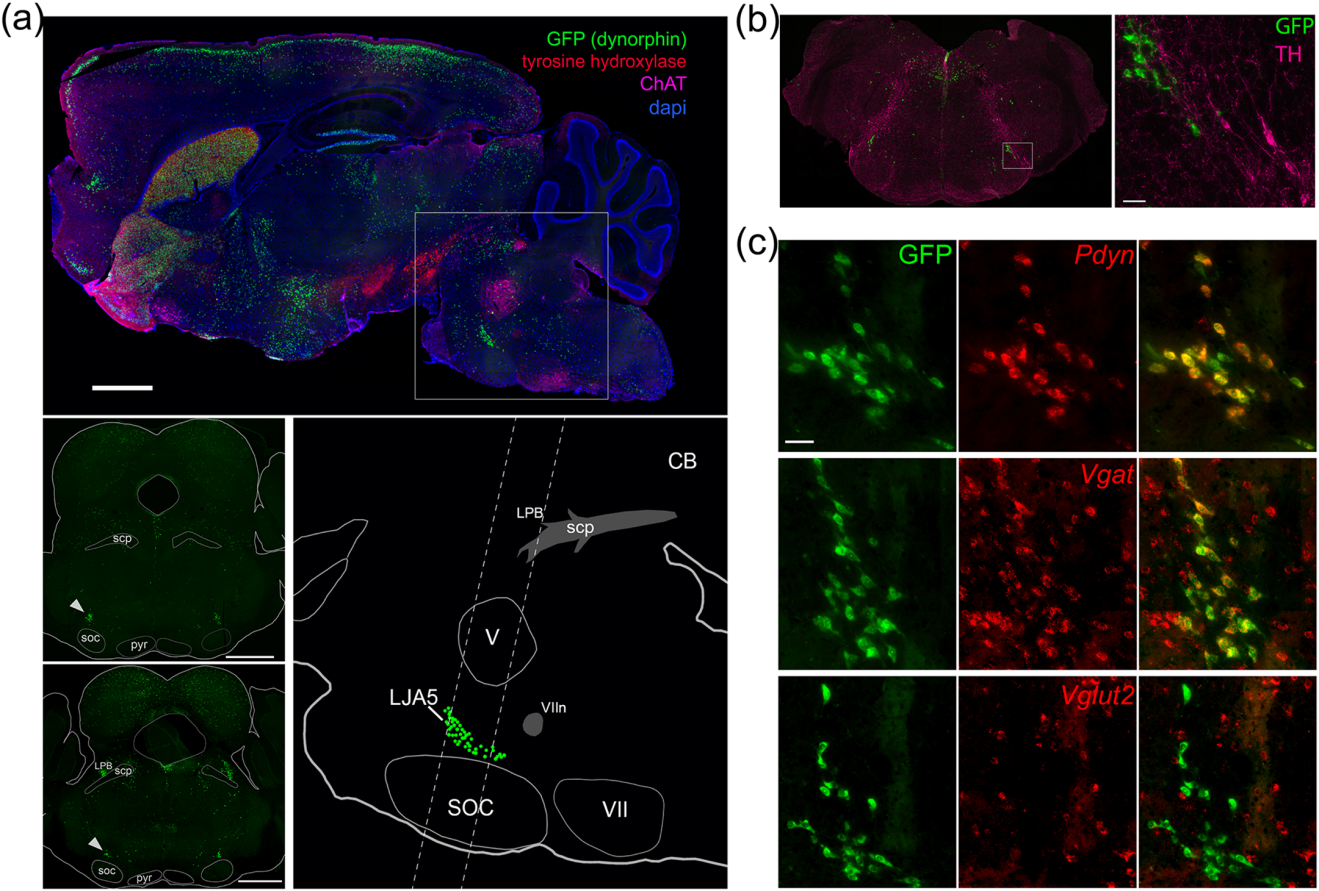
LJA5 is a novel population of prodynorphin‐expressing, GABAergic neurons in the ventrolateral pons. (a) Sagittal section through a *Pdyn‐*green fluorescent protein (GFP) mouse. LJA5 neurons (GFP) are a cluster of prodynorphin neurons in the caudal ventrolateral pons, dorsal to the superior olivary complex (SOC) and rostral to the facial nerve (VIIn). Dotted lines indicate the levels of the coronal sections shown at left. LJA5 is marked by arrowheads (scale bars are 1 mm). (b) Coronal section cut at a steep angle to reveal LJA5 GFP+ neurons in the same section as more caudal A5 tyrosine hydroxylase (TH) expressing neurons. LJA5 neurons do not contain TH (scale bar is 50 μm). (c) In situ hybridization in *Pdyn*‐GFP mouse tissue reveals that all GFP+ neurons colocalize (yellow) with mRNA for prodynorphin (*Pdyn)* and the vesicular transporter for GABA (Vgat, *Slc32a1)*, but not vesicular transporter for glutamate (Vglut2, *Slc17a6)*. (scale bar is 50 μm)

We discovered prodynorphin‐expressing neurons in the rostral, ventrolateral hindbrain (Figure 1(a)), which were not reported previously (Fallon & Leslie, 1986; Merchenthaler et al., 1997). We named this population LJA5 to describe their location in the lateral pons, juxta A5 (noradrenergic cell group). LJA5 neurons lie dorsal to the superior olivary complex. They are also immediately rostral to A5 and the exiting facial nerve root (Figure 1(a)). LJA5 neurons are separate from the catecholaminergic group A5 as they do not express TH (Figure 1(b)). We discovered these neurons in Cre‐reporter mice (*Pdyn‐*GFP) made by crossing a prodynorphin‐Cre mouse with an L10‐GFP reporter mouse (*see Methods)* so that all neurons that have expressed prodynorphin will constitutively express GFP. We confirmed that LJA5 GFP+ neurons express *Pdyn* mRNA (the precursor for dynorphin, an inhibitory opioid neuropeptide) (Figure 1(c)). In this region, 96.7% of the GFP+ neurons express *Pdyn*, and 94.2% of the *Pdyn*+ neurons were also GFP+. Additionally, all LJA5 GFP+ neurons express both markers associated with inhibitory neurotransmission; they express *Slc32a1* (Vgat, vesicular transporter for GABA) and *Gad1* (the enzyme that catalyzes the synthesis of GABA, Agostinelli et al. (2020)). Conversely, none of the LJA5 GFP+ neurons express *Slc17a6* (Vglut2, vesicular transporter for glutamate) (Figure 1(c)).

**FIGURE 2:**
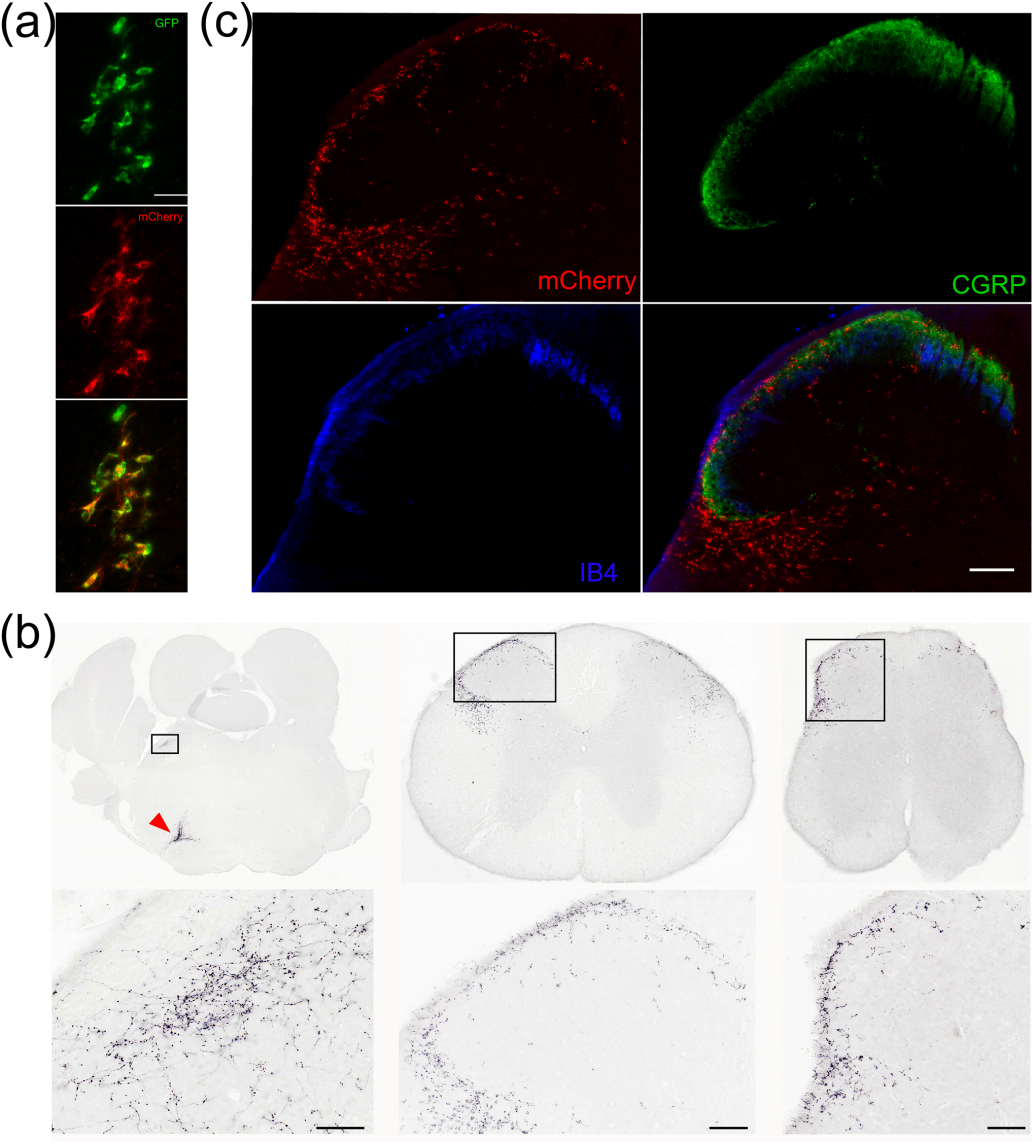
LJA5 axons selectively target sensory regions. (a) A representative injection site of AAV‐DIO‐synaptophysin‐mCherry into LJA5 of this *Pdyn*‐GFP mouse shows that mCherry expression is limited to green fluorescent protein (GFP)‐expressing neurons (scale bar is 50 μm). (b) Synaptophysin‐mCherry densely labels presynaptic boutons throughout lamina I and the lateral spinal nucleus of the entire spinal cord, including upper thoracic (middle) and sacral (right) levels, and in the lateral parabrachial nucleus (left). LJA5 source neurons in the injection site are marked by a red arrow in the ventral brainstem of the first section (scale bars are 50 μm). (c) LJA5 boutons (mCherry+) more densely target lamina I (marked by CGRP peripheral axons) than lamina II (marked by IB4) (scale bar is 100 μm)

To investigate the efferent projections of these inhibitory neurons, we injected a Cre‐dependent anterograde tracer, AAV‐DIO‐synaptophysin‐mCherry, into the LJA5 region of *Pdyn‐*GFP mice (Figure 2(a)). Four weeks later, we analyzed the brain and spinal cord for mCherry‐labeled axons and presynaptic boutons, and found dense terminal fields throughout lamina I of the entire spinal cord and spinal trigeminal nucleus (although sparse labeling is scattered across deeper spinal lamina) (Figure 2(b)).

To confirm that fibers were limited to lamina I, we labeled markers of peripheral sensory axons (CGRP and IB4) that terminate in lamina I or II of the spinal cord, respectively. LJA5 mCherry+ boutons more closely overlapped CGRP+ fibers in lamina I (Figure 2(c)) than IB4+ fibers in lamina II. Besides this extensive, lamina I‐specific projection to the full spinal cord and spinal trigeminal nucleus, we also found LJA5 terminals in lateral spinal nucleus (LSN), and in the brainstem there were small terminal fields in the lateral parabrachial nucleus (PB) and dorsolateral periaqueductal gray (PAG), both of which are implicated in pain and itch (Campos et al., 2018; Gao et al., 2019; Gauriau & Bernard, 2002; Mu et al., 2017; Rodriguez et al., 2017). We quantified the number of mCherry‐labeled boutons in these regions to estimate the relative strength of axonal projections to each target. From these counts, we estimate that LJA5 neurons issue synaptic boutons primarily to the spinal cord (89.9%, specifically 60.2% to lamina I and 29.7% to LSN), with less output to the PAG (5.3%) and PB (4.8%).

### 3.2 Activation of LJA5 neurons inhibits pain and itch behaviors

**FIGURE 3:**
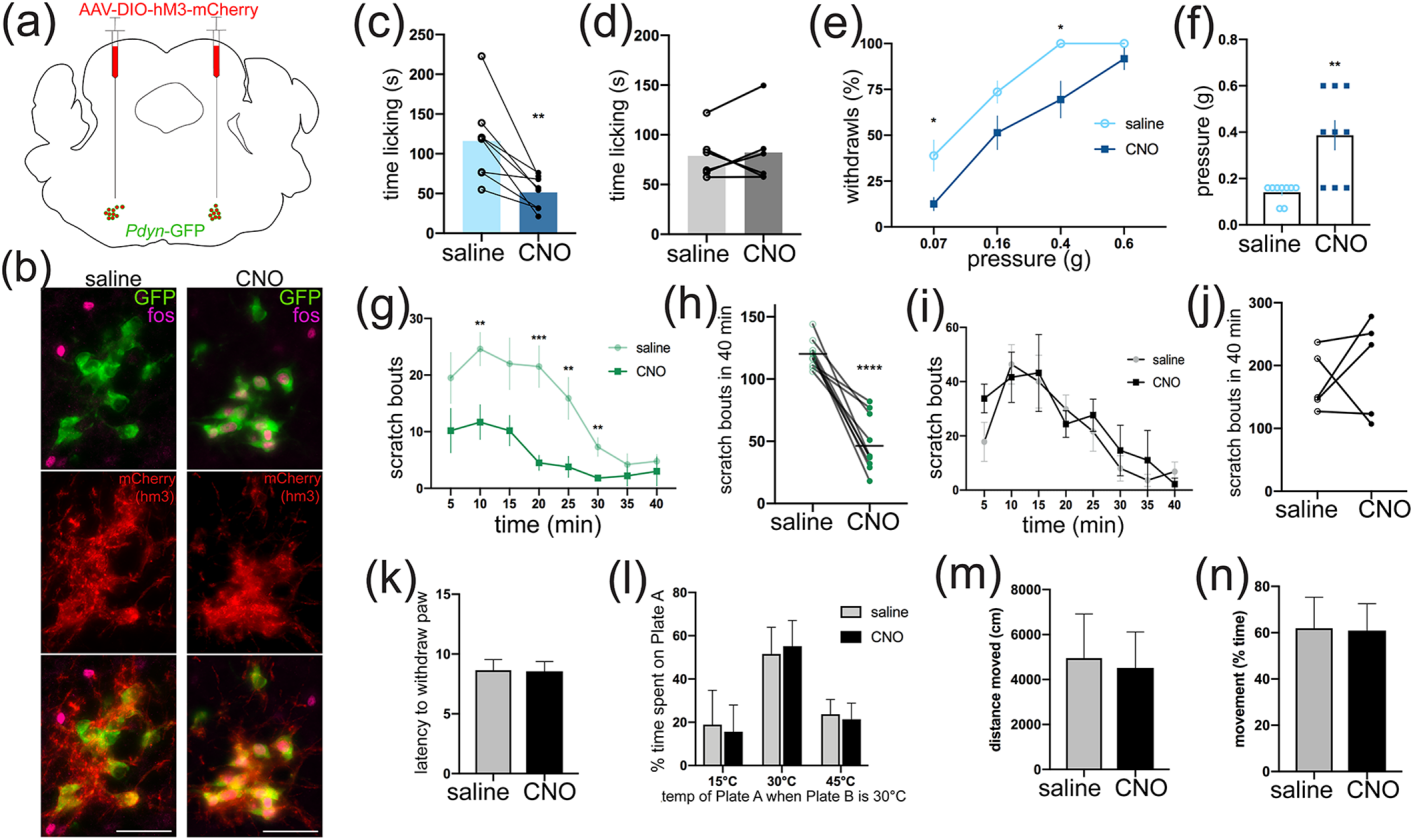
Activating LJA5 neurons selectively suppresses pain and itch behavior. (a) Drawing of AAV‐DIO‐hM3‐mCherry microinjection into LJA5 neurons of a *Pdyn‐*GFP mouse brain. (b) Injection site of AAV‐DIO‐hM3Dq‐mCherry into LJA5 of a *Pdyn‐*green fluorescent protein (GFP) mouse reveals that designer receptor exclusively activated by designer drug (DREADD) expression is limited to GFP‐expressing neurons. Neurons transduced with AAV‐DIO‐hM3Dq‐mCherry express the activity marker Fos after i.p. injection of clozapine‐N‐oxide (CNO) but not saline (scale bar is 50 μm). (c) Effect of CNO (DREADD‐ligand, 1 mg/kg i.p.) or saline on capsaicin‐induced pain behavior (*n* = 9 *Pdyn*‐GFP mice with bilateral AAV‐DIO‐hM3Dq‐mCherry injections into LJA5). Mean for saline (116.2 ± 18.3) compared to CNO (51.4 ± 7.4) conditions; *t* = 4.018, *p* = .0051. (d) CNO had no effect on capsaicin‐induced pain behavior compared to saline injection in *Pdyn*‐GFP mice without DREADD (*n* = 6 *Pdyn*‐GFP mice without AAV‐DIO‐hM3Dq‐mCherry injection). (e) Effect of CNO vs saline on percent of paw withdrawals to von Frey filaments 1.5 h after capsaicin; 0.07 g: *p* = .0057, 0.4 g: *p* = .001526. (f) Effect of CNO vs saline on threshold level (>50% paw withdrawals) to von Frey filaments 1.5 h after capsaicin; t = 4.440, *p* = .0022. (g,h) Effect of CNO (DREADD‐ligand, 1 mg/kg i.p.) or saline on histamine‐induced scratching (*n* = 10 *Pdyn*‐GFP mice with bilateral AAV‐DIO‐hM3Dq‐mCherry injections into LJA5). Scratching bouts are analyzed in 5‐min bins (g) or summed for a total of 40 min (h). (g) **p = .007 (10 min),***p < .0004 (20 min), **p < .009 (25 min), **p = .006 (30 min), two‐way analysis of variance with Holm–Sidak's multiple comparison test. (h) Mean for saline (119.8 ± 3.5) compared to CNO (47.4 ± 7.0) conditions; *t* = 8.7, ****p < .0001. (i,j) CNO had no effect on histamine‐induced scratching in *Pdyn*‐GFP mice without DREADD (*n* = 5 *Pdyn*‐GFP mice without AAV‐DIO‐hM3Dq‐mCherry injections). (k) CNO activation of LJA5 neurons had no effect on latency of paw withdraw in the Hargreaves radiant‐heat test (*n* = 7). (l) CNO activation of LJA5 neurons had no effect on behavioral temperature preference (*n* = 7). Y‐axis is the percent of time spent on Plate A when Plate B is 30°C. (m,n) CNO activation of LJA5 had no effect on ambulation (*n* = 7)

Lamina I neurons transmit pain, temperature, and itch (Craig, 2002; Dong & Dong, 2018) so we hypothesized that LJA5 neurons inhibit these sensory modalities. To test this hypothesis, we chemogenetically activated these neurons using a Cre‐dependent DREADD (AAV‐DIO‐hM3Dq‐mCherry). Intraperitoneal (i.p.) injection of CNO binds to the hM3Dq receptor on the *Pdyn*‐Cre‐expressing neurons and activates them through the Gq‐signaling cascade. After behavioral tests, we counted the number of LJA5 GFP+ neurons that were transduced with AAV‐DIO‐hM3Dq‐mCherry in each mouse. To confirm that CNO activates hM3Dq‐expressing neurons, we counted the number of transduced cells (mCherry+) expressing an activity marker, Fos, after injecting saline or CNO. Ninety minutes after injection, 1.9% ± 0.3 (saline) or 85.3% ± 1.4 (CNO) of LJA5 neurons with mCherry also expressed Fos (Figure 3(b)).

We then tested the effects of activating LJA5 neurons on pain, itch, and temperature sensation. The mice were used as their own controls, with i.p. injection of saline or CNO on different days, 30 min before each test. Activating LJA5 neurons with CNO markedly suppressed capsaicin‐induced pain behavior and mechanical hypersensitivity (Figure 3(c,e,f)). CNO also reduced histamine‐induced scratching (Figure 3(g,h)). To ensure that CNO itself is not analgesic or anti‐pruritic, we made i.p. injections of saline or CNO in *Pdyn*‐GFP mice without AAV‐DIO‐hM3Dq‐mCherry, and we saw no differences in pain or itch behavior (Figure 3(d,i,j)). Finally, in contrast to the large reductions in pain and itch‐associated behaviors, DREADD activation of LJA5 neurons did not alter temperature preference, reflexive thermal pain, or ambulation (Figure 3(k–n)). These results indicate that LJA5 neurons specifically suppress pain and itch without altering thermosensory or motor functions.

## 4 DISCUSSION

**FIGURE 4:**
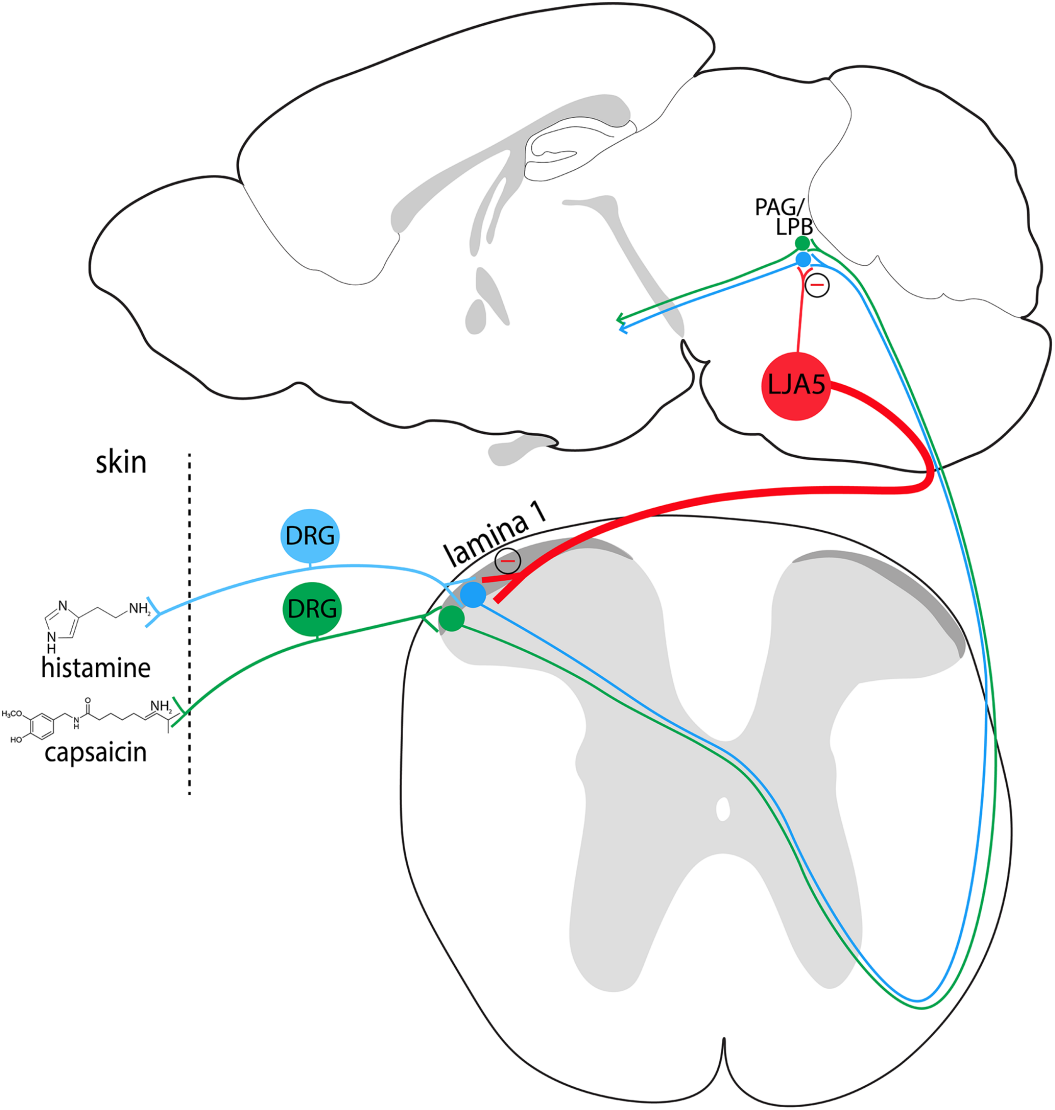
Inhibition of pain and itch transmission by LJA5 neurons. Histamine and capsaicin in the skin are detected by receptors on DRG nerve fibers that transmit pain and itch information to the spinal cord and up to the brain. LJA5 *Pdyn*‐expressing GABAergic neurons in the brainstem send inhibitory descending projections to sensory relay neurons in lamina I of the dorsal horn, lateral parabrachial nucleus (LPB), and periaqueductal gray (PAG). Activating this inhibitory “top‐down” projection suppresses pain and itch signaling

The idea that our brains can suppress unpleasant sensations began with a report in 1969 that electrically stimulating the brainstem in rats could produce a surgical level of anesthesia, without anesthetic drugs (Reynolds, 1969). A roadblock to developing this observation is that the stimulated region of the brainstem does not send any inhibitory projections to the spinal cord. Additionally, no lamina I‐selective projection was identified until now. Here, we identify a group of prodynorphin‐expressing GABAergic brainstem neurons with specific inhibitory projections to lamina I of the spinal cord. We have previously investigated the inputs and outputs of this group (Agostinelli et al., 2020), and here we further explore the efferent spinal projections. We further show that chemogenetic activation of these neurons reduces pain and itch, while sparing temperature preference or reflexive thermal responses. Inhibition by LJA5 neurons updates current models of pain and itch circuitry (Figure 4).

### 4.1 Characterizing LJA5 neurons

Previously, the only known neuronal population in this region of the brainstem was the noradrenergic cell group, A5. A5 neurons also project to the spinal cord, but their projection to deep autonomic laminae is distinct from the lamina I‐targeted projections of prodynorphin‐expressing LJA5 neurons (Bruinstroop et al., 2012; Loewy et al., 1979). Also, LJA5 neurons are distributed rostral to A5 neurons, with the tail end of LJA5 neurons tapering medial to the facial nerve root exit, where rostral A5 neurons are located. Additionally, LJA5 neurons lack the catecholamine‐synthesizing enzyme TH, which is expressed by A5 neurons (Figure 1(b)). Therefore, to describe their location in relation to A5, we named this population LJA5 as it sits in the lateral pons, juxta A5. We have additionally shown that *Pdyn*+ neurons in a similar location in human brainstems (Agostinelli et al., 2020).

We discovered these prodynorphin‐expressing neurons in a *Pdyn‐*GFP reporter mouse and showed that these GFP‐expressing neurons express *Pdyn* mRNA (Figure 1(c)).

Previous studies in the rat brain did not comment on these neurons, although neurons containing *Pdyn* mRNA or dynorphin immunoreactivity can be seen above the superior olive (where we see LJA5 neurons in mice) in their figures (Fallon & Leslie, 1986; Merchenthaler et al., 1997).

Additionally, LJA5 neurons are both dynorphinergic and GABAergic (Figure 1(c), (Agostinelli et al., 2020). Whether the antipruritic and analgesic effects of activating LJA5 neurons are mediated by dynorphin, GABA, or another neurotransmitter remains unknown. Dynorphin is an endogenous, inhibitory opioid peptide located in many regions of the forebrain, brainstem, and spinal cord (Merchenthaler et al., 1997). Four identified opioid receptors respond to at least four different endogenous peptide ligands (Waldhoer et al., 2004). Dynorphin peptides derive from the precursor prodynorphin, and bind to the kappa opioid receptor, which is expressed by neurons in many regions, including lamina I of the spinal cord (Harris et al., 2004; Snyder et al., 2018; Waldhoer et al., 2004). Opioid agonists that bind to the mu opioid receptor (including morphine) are most commonly used for analgesic treatment, but these agonists have unpleasant and serious side effects including pruritis, constipation, and respiratory suppression (Chaney, 1995). Worse, due to their development of tolerance, mu opioid agonists are highly addictive (Waldhoer et al., 2004), with drugs like heroin and fentanyl contributing to an increasing epidemic of drug overdose deaths (Rudd et al., 2016). More recently, kappa opioid agonists have been shown to be antipruritic (Inan & Cowan, 2004; Kardon et al., 2014; Kumagai et al., 2012; Wikstrom et al., 2005) and analgesic (Ansonoff et al., 2006; Kivell & Prisinzano, 2010) in mice and humans, and may be a safer alternative to mu opioid agonists due to their low addictive potential and lack of respiratory depression.

In mice, intrathecal administration of kappa‐opioid agonists reduces itch (Kardon et al., 2014). Initially, this finding led to the suggestion that dynorphin released by interneurons in the spinal cord inhibits itch (Kardon et al., 2014). Our discovery that dynorphinergic neurons in the brainstem project to lamina I of the spinal cord, and that activating these neurons inhibits pain and itch, suggests LJA5 as a key source of dynorphin for kappa‐opioid inhibition of pain and itch sensory transmission. Our finding that LJA5 neurons can reduce both itch and pain holds immense therapeutic potential. They represent the first known source of dynorphinergic projections from the brain to the spinal cord for itch and pain modulation, and the first known GABAergic projection that selectively targets lamina I. LJA5 neurons therefore are a promising target for understanding endogenous modulation of noxious sensory circuits and treating patients with chronic pruritis or pain.

### 4.2 Spinal projections

Perhaps the most remarkable feature of this group of neurons is that they are the first and only known group of inhibitory neurons that provide direct axonal projections selectively targeting lamina I of the spinal cord, whose neurons transmit pain, itch, and thermal sensory information from the skin. Outside lamina I, the only substantial LJA5 terminals we find in the spinal cord target the adjoining LSN, a loose collection of neurons in the dorsolateral funiculus. LSN neurons receive nociceptive input via primary afferents, similar to lamina I, but from subcutaneous muscle and visceral tissues (Neuhuber, 1982; Neuhuber et al., 1986; Sikandar et al., 2017). Thus, LJA5 projections to the spinal cord are well positioned to inhibit the full suite of noxious sensory information transmitted through the spinal cord to the brain.

In contrast to the selective LJA5 projection to lamina I and the LSN, previously identified descending projections from the brainstem nonspecifically target multiple spinal laminae. A limited number of brainstem populations send descending projections to the spinal cord, and previous work on top‐down pathways focused on bulbospinal neurons in the rostral ventromedial medulla (RVM), which project serotonergic and GABAergic axons nonspecifically across multiple laminae of the spinal gray matter (Basbaum et al., 1978; Fields et al., 1991; Millan, 2002). Additionally, the locus coeruleus sends noradrenergic projections to all laminae of the full spinal cord (Bruinstroop et al., 2012; Clark & Proudfit, 1991). These neurons are thought to mediate anti‐nociceptive effects that can be modulated pharmacologically with anti‐depressant medications (serotonin and norepinephrine reuptake inhibitors) sometimes used clinically to treat chronic neuropathic pain (Kremer et al., 2016). However, these drugs only partially reduce pain symptoms and are not effective in treating all types of pain (Gilron, 2016), suggesting that there are additional antinociceptive mechanisms.

Outside the brainstem, a small number of hypothalamic neurons also project axons to the spinal cord, without specifically targeting lamina I. A subset of neurons in the paraventricular hypothalamic nucleus sends axons to the IML and multiple laminae including I, II, and X (Saper et al., 1976; Swanson & McKellar, 1979). Orexin neurons of the lateral hypothalamus project to the spinal trigeminal nucleus (Peyron et al., 1998) and several laminae of the spinal cord, including I, X, and the IML (van den Pol, 1999). However, while orexin neurons have been implicated in reward and wakefulness, they have not been implicated in the top‐down modulation of pain or itch sensation at the level of the spinal cord.

It should also be noted that the pain has been discussed as eliciting multidimensional responses, with emphasis on the reflexive verses affective/coping responses to pain (Fields, 1999; Han et al., 2015; Huang et al., 2019). Our initial studies suggest LJA5 neurons might play a larger role in modulating affective pain responses (licking post‐capsaicin and subsequent mechanical hypersensitivity) compared to reflexive responses (radiant heat). One study found that *Tac1*+ lamina I projection neurons are necessary for affective (coping or motivation) pain responses (Huang et al., 2019). We previously showed that LJA5 neurons did not appear to preferentially target these *Tac 1*+ neurons (Agostinelli et al., 2020), so perhaps LJA5 neurons are involved in affective pain through other lamina I neurons, or higher up in this pathway such as through the PB.

### 4.3 Supraspinal projections

Identifying this new pathway for inhibiting itch and pain raises new questions. For example, relative to their extensive projections to lamina I, what is the role of LJA5 collateral projections to the PB and PAG? Given that neurons in both regions are major, downstream targets for nociceptive and pruritic neurons in lamina I, the simplest hypothesis is that LJA5 collaterals to PB and PAG simply inhibit downstream sensory‐relay neurons in parallel with the primary site of inhibition, upstream in lamina I. We found that roughly 90% of LJA5 boutons are in the spinal cord (compared to 5% in each the PAG and PB), suggesting that lamina I neurons are the primary synaptic targets through which LJA5 neurons suppress itch and pain. However, future studies should determine the function of LJA5 neurons in the spinal cord verses the PB and PAG.

Traditionally, sensory information was thought to be transmitted from the periphery to the brain via the spinothalamic pathway. However, multiple studies have shown that lamina I neurons of the spinal cord directly project to the PB (Cechetto et al., 1985; Craig, 1995; Panneton & Burton, 1985), comprising the spinoparabrachial pathway. Additional studies confirmed connectivity between nociceptive‐specific lamina I neurons and PB neurons (Bester et al., 2000; Light et al., 1993). Furthermore, PB neurons respond to pain (Campos et al., 2018), and PB inhibition is both antinociceptive (Han et al., 2015; Rodriguez et al., 2017) and antipruritic (Campos et al., 2018; Mu et al., 2017). Since LJA5 neurons send inhibitory projections to the PB, future studies will help clarify the role of LJA5 in modulating sensory transmission in the PB.

Studies on the descending control of pain have focused on the PAG projection to the RVM, which projects nonspecifically across the spinal cord (Fields et al., 1991; Millan, 2002). Additionally, there are direct projections from lamina I of the spinal cord to the PAG (Craig, 1995), implicating the PAG in both nociceptive reception and descending modulation. The PAG has mixed effects on pain and itch sensation; PAG stimulation induces analgesia (Reynolds, 1969; Yeung et al., 1977) and scratching behavior (Gao et al., 2019), and inhibiting PAG neurons reduces scratching in mice (Gao et al., 2019). Given this heterogeneity among PAG neurons in regards to nociception, further studies are required to clarify the functional relationship between LJA5 and PAG neurons.

LJA5 inhibitory axonal projections to the spinal cord are the first and only known top‐down pathway by which the brain may directly and selectively inhibit pain and itch. This discovery represents a major development in our basic understanding of how the brain not only receives, but modifies noxious sensory information. Given that the LJA5 region receives input from many stress‐related forebrain structures (Agostinelli et al., 2020), we suspect that descending stress signals activate LJA5 neurons, which would then in turn inhibit pain or itch. This phenomenon is known as stress‐induced analgesia, and it might be evolutionarily beneficial animal can focus on behavioral responses during an acutely noxious situation without much distraction from pain or itch (Amit & Galina, 1988). Inhibition by LJA5 neurons updates current models of pain and itch circuitry and opens the possibility of novel analgesic and anti‐pruritic therapeutics for patients with chronic pain and pruritis.

## CONFLICT OF INTEREST

The authors declare no conflict of interest.

## AUTHOR CONTRIBUTIONS

**Lindsay J Agostinelli**: Identified the novel neurons and performed mouse surgeries, histology, immunostaining, and behavior experiments. **Lindsay J. Agostinelli**: Reviewed and analyzed data with **Alexander G. Bassuk**. **Lindsay J. Agostinelli**: Prepared figures and wrote the manuscript. **Lindsay J. Agostinelli**: and **Alexander G. Bassuk**: Edited and approved the final submitted version.

### PEER REVIEW

The peer review history for this article is available at https://publons.com/publon/10.1002/cne.25076.

## Data Availability

The data that support the findings of this study are available from the corresponding author upon reasonable request.
